# Small molecules targeting coxsackievirus A16 capsid inactivate viral particles and prevent viral binding

**DOI:** 10.1038/s41426-018-0165-3

**Published:** 2018-09-26

**Authors:** Chien-Ju Lin, Ching-Hsuan Liu, Jonathan Y. Wang, Chun-Ching Lin, Yi-Fang Li, Christopher D. Richardson, Liang-Tzung Lin

**Affiliations:** 1 School of Pharmacy, College of Pharmacy, Kaohsiung Medical University, Kaohsiung, 80708 Taiwan; 2 Graduate Institute of Medical Sciences, College of Medicine, Taipei Medical University, Taipei, 11031 Taiwan; 3 Department of Microbiology and Immunology, Dalhousie University, Halifax, Nova Scotia B3H 4R2 Canada; 4 Department of Molecular Biosciences, The University of Texas at Austin, Austin, TX 78712 USA; 5 Graduate Institute of Natural Products, College of Pharmacy, Kaohsiung Medical University, Kaohsiung, 80708 Taiwan; 6 Department of Pediatrics and Canadian Center for Vaccinology, Izaak Walton Killam Health Centre, Halifax, Nova Scotia B3K 6R8 Canada; 7 Department of Microbiology and Immunology, School of Medicine, College of Medicine, , Taipei Medical University, Taipei, 11031 Taiwan

## Abstract

Coxsackievirus A16 (CVA16) is an etiologic agent of hand, foot, and mouth disease (HFMD) that affects young children, and although typically self-limited, severe complications, and fatal cases have been reported. Due to the lack of specific medication and vaccines against CVA16, there is currently a need to develop effective antivirals to better control CVA16 infections in epidemic areas. In this study, we identified the tannins chebulagic acid (CHLA) and punicalagin (PUG) as small molecules that can efficiently disrupt the CVA16 infection of human rhabdomyosarcoma cells. Both compounds significantly reduced CVA16 infectivity at micromolar concentrations without apparent cytotoxicity. A mechanistic analysis revealed that the tannins particularly targeted the CVA16 entry phase by inactivating cell-free viral particles and inhibiting viral binding. Further examination by molecular docking analysis pinpointed the targets of the tannins in the fivefold axis canyon region of the CVA16 capsid near the pocket entrance that functions in cell surface receptor binding. We suggest that CHLA and PUG are efficient antagonists of CVA16 entry and could be of value as antiviral candidates or as starting points for developing molecules to treat CVA16 infections.

## Introduction

Hand, foot, and mouth disease (HFMD) is a common illness in young children that includes symptoms such as maculopapular or vesicular rashes on the soles, palms and buttocks and oral ulcers in the pharynx^1^. The two major causative agents of HFMD are coxsackievirus A16 (CVA16) and enterovirus 71 (EV71)^2^. During the last two decades, large-scale HFMD outbreaks have occurred due to CVA16 in the Asia-Pacific region, including in Taiwan^3^, Singapore^4^, and China, where they have resulted in many severe cases and fatalities^5,6^. Although clinical symptoms and disease caused by CVA16 infection are typically milder than those caused by EV71^7^, CVA16 infections have been reported to have more severe complications, such as brainstem encephalitis, aseptic meningitis, paralysis, myelitis, myocarditis and pericarditis, acute heart failure, and even fatal pneumonitis^8,9^. Importantly, no licensed antiviral therapy or vaccine currently exists against CVA16, highlighting the need to develop-specific antiviral strategies for the management of future outbreaks.

CVA16 belongs to the *Enterovirus* genus of the *Picornaviridae* family, which also includes EV71. The CVA16 particle is small (diameter ~30 nm) and non-enveloped, and its 7.4 kb positive single-strand RNA genome generates a large polyprotein that is divided into consecutive P1, P2, and P3 parts. The P2 and P3 regions consist of non-structural proteins associated with viral replication, whereas processing of P1 yields 4 structural proteins (VP1-4) that associate into pentamers and self-assemble to form the viral icosahedral capsid. VP1, VP2, and VP3 are located at the surface of the capsid, whereas VP4 is an internal protein^5^. Similar to many enteroviruses, the CVA16 virion shows a depression encircling the fivefold axis (also called the ‘canyon’) on its surface that is responsible for receptor binding^10^. Both the human P selectin glycoprotein ligand-1 (PSGL-1) and scavenger receptor class B member 2 (SCARB2) have been suggested to be cellular receptors for CVA16^11–13^. At the canyon floor is a hydrophobic pocket within the VP1 capsid protein that binds natural lipids (termed ‘pocket factors’)^10^. Expulsion of this fatty acid molecule during receptor binding is a prerequisite to conformational change of the virion capsid, which results in the externalization of the amino termini of VP1 and VP4 and forms a channel in the membrane. The viral genome is subsequently ejected through the channel and enters the cell cytoplasm for replication, with completion of the viral life cycle ending with release of mature viral particles upon cell lysis.

Many natural products, including tannins, flavonoids, and saponins have been demonstrated to possess antiviral activities^14^. More importantly, several have been documented to exert an inhibitory effect against viral entry, including gallic acid and saikosaponin b2 against hepatitis C virus (HCV)^15^, (-)-epigallocatechin-3-gallate against Zika virus^16^, and the *Ganoderma lucidum* triterpenoids lanosta-7,9(11),24-trien-3-one,15,26-dihydroxy and ganoderic acid Y against EV71^17^. These observations demonstrate that natural products are an excellent source of antiviral drugs and set a precedent for our study.

In an attempt to develop antivirals against CVA16, we screened a number of natural product classes of compounds and identified two tannins, chebulagic acid (CHLA), and punicalagin (PUG), as efficient inhibitors of CVA16 entry. We further determined the polar contacts of the tannins on the CVA16 capsid, which were specifically concentrated in the fivefold axis depression region known to mediate CVA16-receptor interactions. We suggest that CHLA and PUG may be of value as starting points for the development of a therapeutic agents against CVA16.

## Results

### Identification of two tannins with antiviral activity against CVA16

**Fig. 1:**
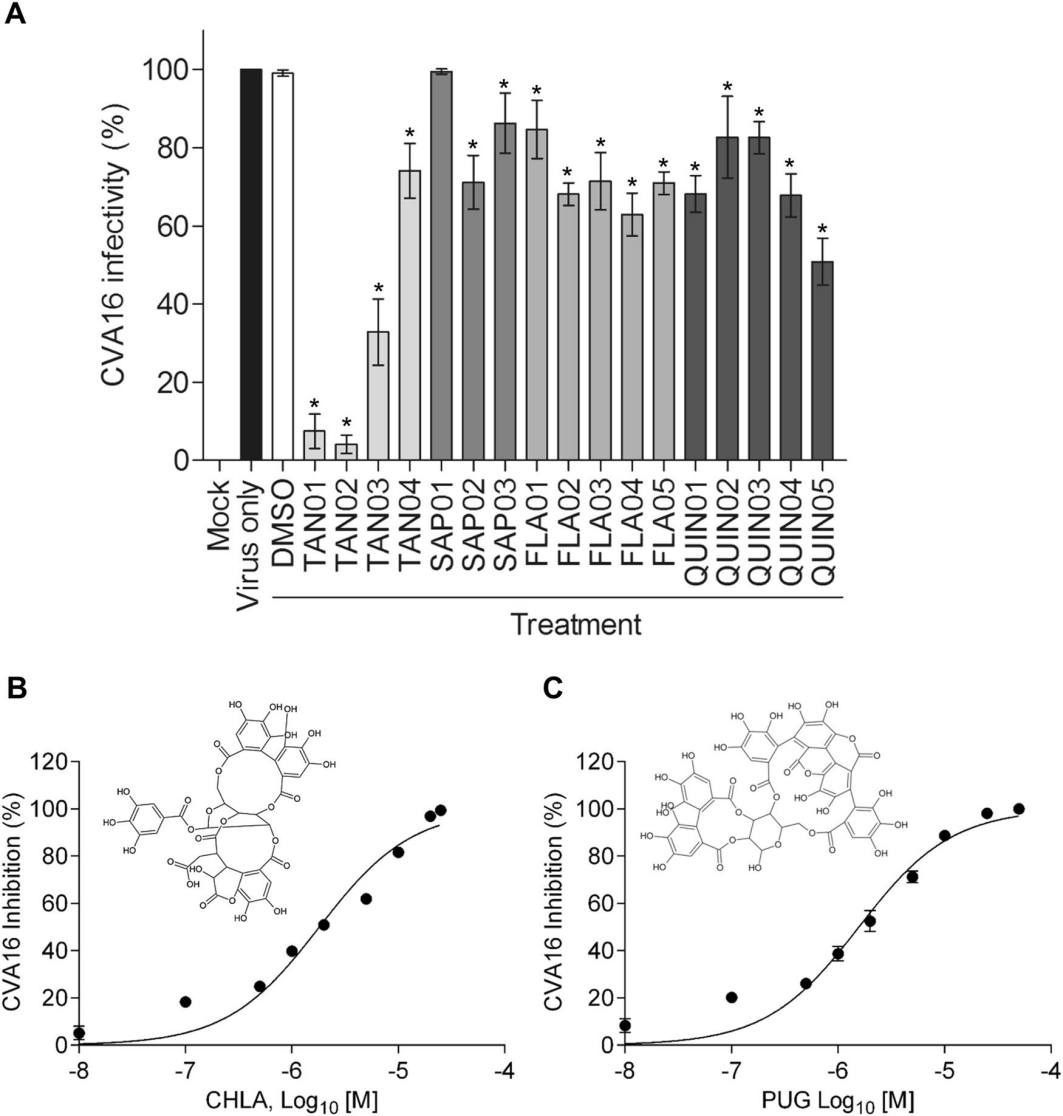
Drug screening analysis and antiviral dose-response activity of CHLA and PUG against CVA16. **a** RD cells were challenged with CVA16 (50 PFU/well) in the presence of the test compounds and then analyzed by a plaque assay 72 h post infection; the results are expressed as percent (%) CVA16 infectivity. **b**, **c** RD cells were infected with CVA16 (50 PFU/well) in the presence of **b** CHLA (0.01, 0.1, 0.5, 1, 2, 5, 10, 20, and 25 µM) or **c** PUG (0.01, 0.1, 0.5, 1, 2, 5, 10, 25, and 50 µM) and analyzed for virus-induced plaque formation after 72 h of incubation; the data are expressed as % CVA16 inhibition. Chemical structures of CHLA and PUG are shown in the respective panels. For all experiments, a DMSO (0.25%) treatment served as negative control, and the data shown are the means ± standard deviation (SD) from three independent repeats. *p < 0.05 compared to the ‘virus only’ group

Different classes of natural products, including tannins, triterpenes, flavonoids, quinones, and their derivatives (Table 1), were screened for their inhibitory activity against CVA16 infection. Before assessing their antiviral effects, the cytotoxicity of these compounds was determined in human rhabdomyosarcoma (RD) cells using an XTT cell viability assay (data not shown). The maximum subcytotoxic dose of each compound was chosen for the evaluation of their antiviral activity against CVA16 using an antiviral plaque assay. As shown in Fig. 1a, only the two tannins CHLA (TAN01; 20 µM) and PUG (TAN02; 25 µM) significantly inhibited CVA16 infectivity by >90% among all the tested compounds. Therefore, CHLA and PUG were investigated for the remainder of the study.

**Table 1:** Test compound classes used in this study

Code	Compound	Class
TAN01	Chebulagic acid	Tannin
TAN02	Punicalagin	Tannin
TAN03	Chebulinic acid	Tannin
TAN04	5-O-galloyl-shikimic acid	Tannin
SAP01	Saikosaponin a	Triterpene
SAP02	Saikosaponin b2	Triterpene
SAP03	Saikosaponin c	Triterpene
FLA01	TCH-3105	Flavonoid
FLA02	TCH-3154	Flavonoid
FLA03	TCH-3188	Flavonoid
FLA04	TCH-3529	Flavonoid
FLA05	TCH-3531	Flavonoid
QUIN01	Q4224	Quinone
QUIN02	Q4236	Quinone
QUIN03	Q4246	Quinone
QUIN04	Q4248	Quinone
QUIN05	Q4250	Quinone

### CHLA and PUG inhibit CVA16 infection in a dose-dependent manner

To determine the antiviral activity of CHLA (Fig. 1b) and PUG (Fig. 1c), we performed a dose-response analysis. Various concentrations of the test compounds were added during CVA16 infection of RD cells. Both tannins were observed to efficiently reduce the cytopathic effect caused by CVA16 infection in a dose-dependent manner. The calculated EC_50_ values of CHLA and PUG were 6.92 ± 0.25 and 6.29 ± 0.49 µM, respectively. Based on the observed cytotoxicity of CHLA (CC_50_ = 45.62 ± 9.58 µM) and PUG (CC_50_ = 76.96 ± 12.07 µM), the determined selectivity index (SI = CC_50_/EC_50_) of the two tannins against CVA16 was determined to be 6.59 (CHLA) and 12.24 (PUG), respectively.

### CHLA and PUG treatments inhibit CVA16 during the early steps of infection and depend on the presence of the virus

**Fig. 2:**
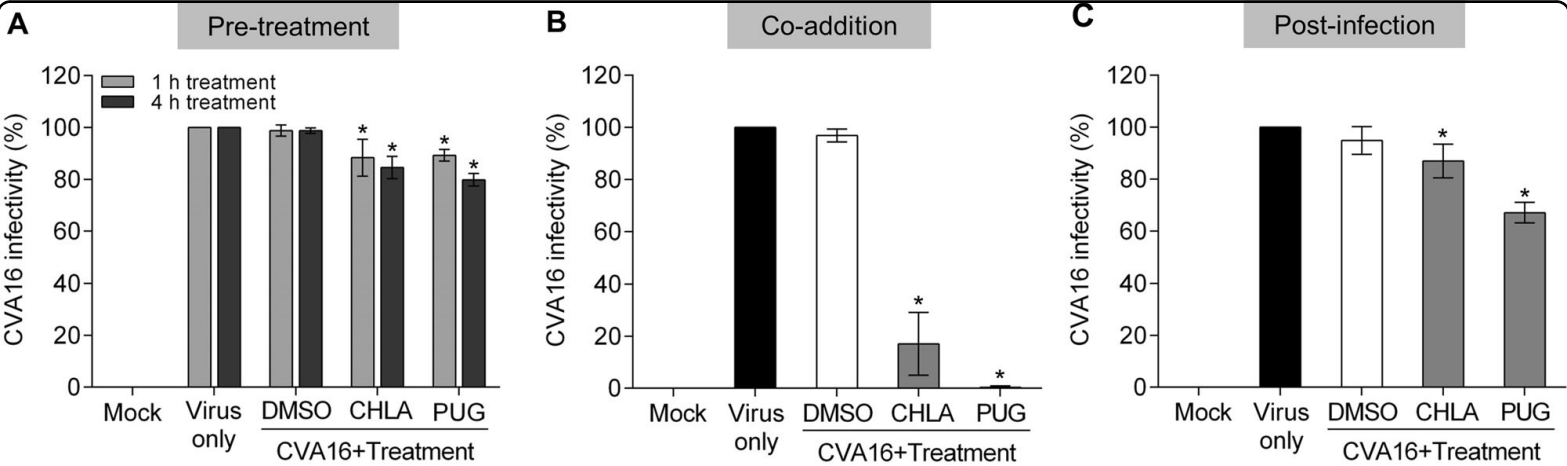
Time-of-drug-addition effect of CHLA and PUG against CVA16 infectivity. RD cells were treated with CHLA (20 µM) or PUG (25 µM) at different times of CVA16 inoculation (50 PFU/well). **a** For the pretreatment, cells were incubated with the test compounds for 1 or 4 h and then were washed before CVA16 infection. **b** For coaddition assays, cells were administered with drugs and virus simultaneously for 1 h and were subsequently washed. **c** In the postinfection experiment, cells were infected with CVA16 for 1 h, washed, and treated with the test compounds. For all of the above experiments, a DMSO (0.25%) treatment was included as negative control for each condition, and viral plaques were stained and counted after 72 h of incubation. The data shown are the means ± SD from three independent experiments. *p < 0.05 compared to the respective ‘virus only’ group

We previously observed that CHLA and PUG inhibit the entry step of a number of viruses^18,19^. To explore the possibility that the anti-CVA16 effect of CHLA and PUG involves disruption of viral entry, we added the two tannins at different times during CVA16 infection, including pre-entry, entry, and postentry. To examine the effect of the treatments on viral pre-entry and on the cells, RD cells were pretreated with CHLA (20 µM) or PUG (25 µM) for 1 or 4 h, and then washed prior to infection by CVA16. To assess the effect on the viral entry stage, RD cells were treated with CVA16 and the tannins simultaneously. To assess the effect of the compounds after viral entry, RD cells were first infected with CVA16 for 1 h before washing and overlaying the cells with media containing the tannins. As shown in Fig. 2, the pretreatment (Fig. 2a) and postinfection (Fig. 2c) treatments with CHLA and PUG had only slight impact in preventing CVA16 infection. However, when the tannins were added concurrently to the viral challenge, they effectively inhibited plaque formation (Fig. 2b). These results indicate that CVA16 infection is only suppressed by these compounds when they are present at the time-of-the infection. Altogether, the above observations suggest that the anti-CVA16 activities of CHLA and PUG are unlikely to be mediated through masking of the CVA16 cellular receptors or by directly affecting the host cells and the postinfection viral replication phase. Rather, they necessitate the presence of the enterovirus and likely target the early steps of its infection.

### CHLA and PUG block CVA16 entry by disrupting viral attachment

**Fig. 3:**
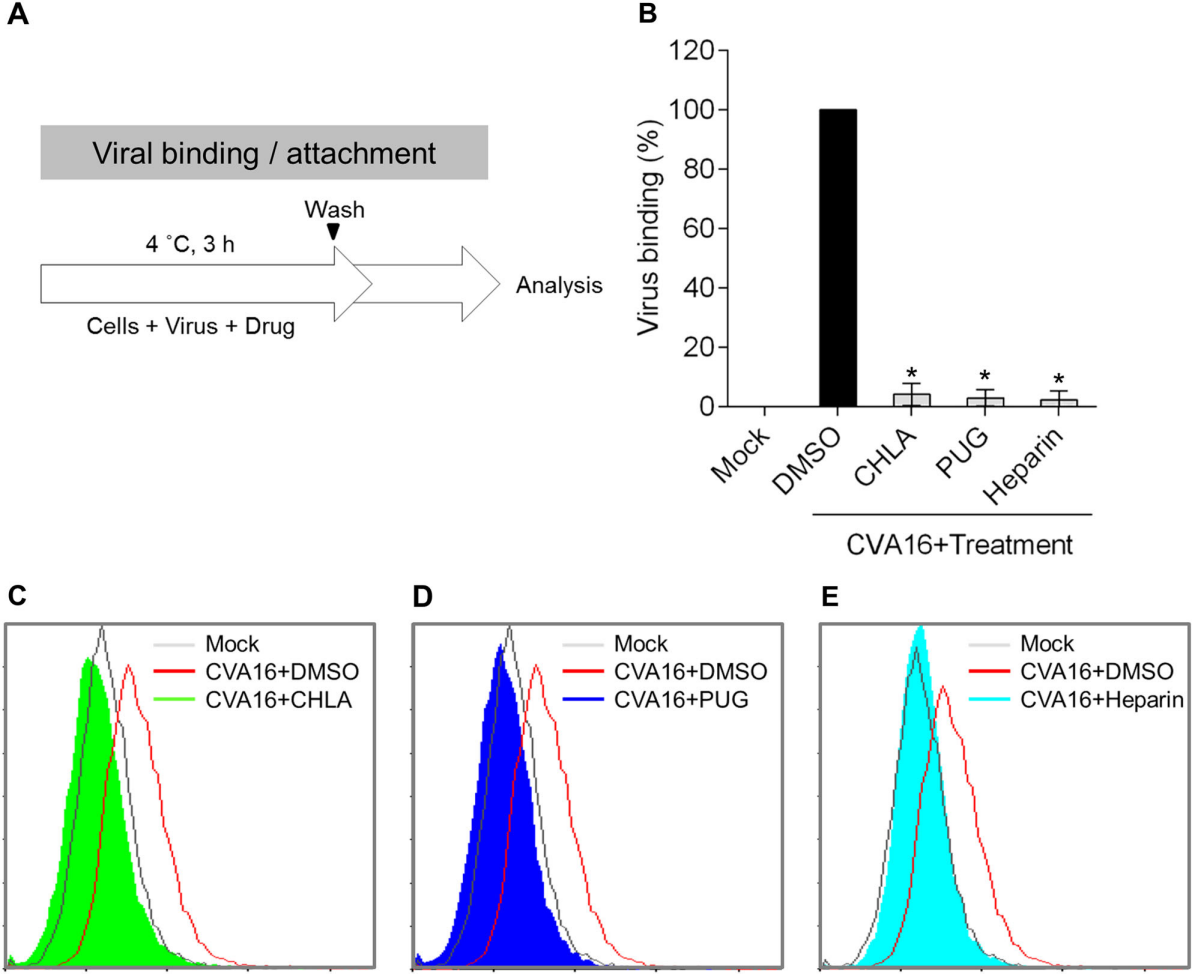
CHLA and PUG abolish CVA16 binding to the host cell. **a** Schematic of the experiment. **b** RD cells (2 × 10^5^ cells/well) were infected with CVA16 (MOI = 100) in the presence or absence of CHLA (20 µM), PUG (25 µM), soluble heparin (500 µg/ml), or DMSO (0.25%, negative control) for 3 h at 4 °C. The inocula were removed and the cells were then collected into tubes, washed twice, fixed, and stained with anti-VP1 antibody followed by an Alexa 488-conjugated secondary antibody for flow cytometry detection of surface-bound viruses. The quantified data from the detected fluorescence signals were plotted as the means ± SD from three independent experiments in bar graph as ‘Viral binding (%)’. *p < 0.05 compared to the ‘DMSO’ control treatment. The representative flow cytometry histograms of CHLA **c**, PUG **d**, and heparin **e** treatments are shown

Based on the above results, we hypothesized that the tannins may inhibit CVA16 infection by targeting the viral entry phase. To elucidate the antiviral mechanism, we next determined the impact of CHLA and PUG against CVA16 attachment to the host cell surface using a flow cytometry-based viral binding assay to detect surface-bound virions^20^. RD cells were prechilled at 4 °C and then infected with CVA16 in the presence or absence of the test compounds on ice (Fig. 3a). Performing this step at 4 °C permits viral binding to cells but precludes viral internalization. The treated cells were subsequently collected, washed, and fixed for VP1 staining and flow cytometry analysis. As shown in the quantitative data in Fig. 3b and in the respective histograms for CHLA (Fig. 3c) and PUG (Fig. 3d), the DMSO treatment had no effect against CVA16 binding, and the virions were readily detected on the RD-cell surfaces. In contrast, both CHLA and PUG effectively disrupted binding of the viral particles to the cell surface membranes, abolishing most, if not all, of the cell-associated VP1 signals. Similar results were obtained with treatment using soluble heparin (Fig. 3b, e), which is known to block CVA16 attachment^21^. This observation suggests that the tannins block CVA16 infection by impeding the viral attachment step.

### CHLA and PUG neutralize CVA16 infectivity by inactivating free viral particles

**Fig. 4:**
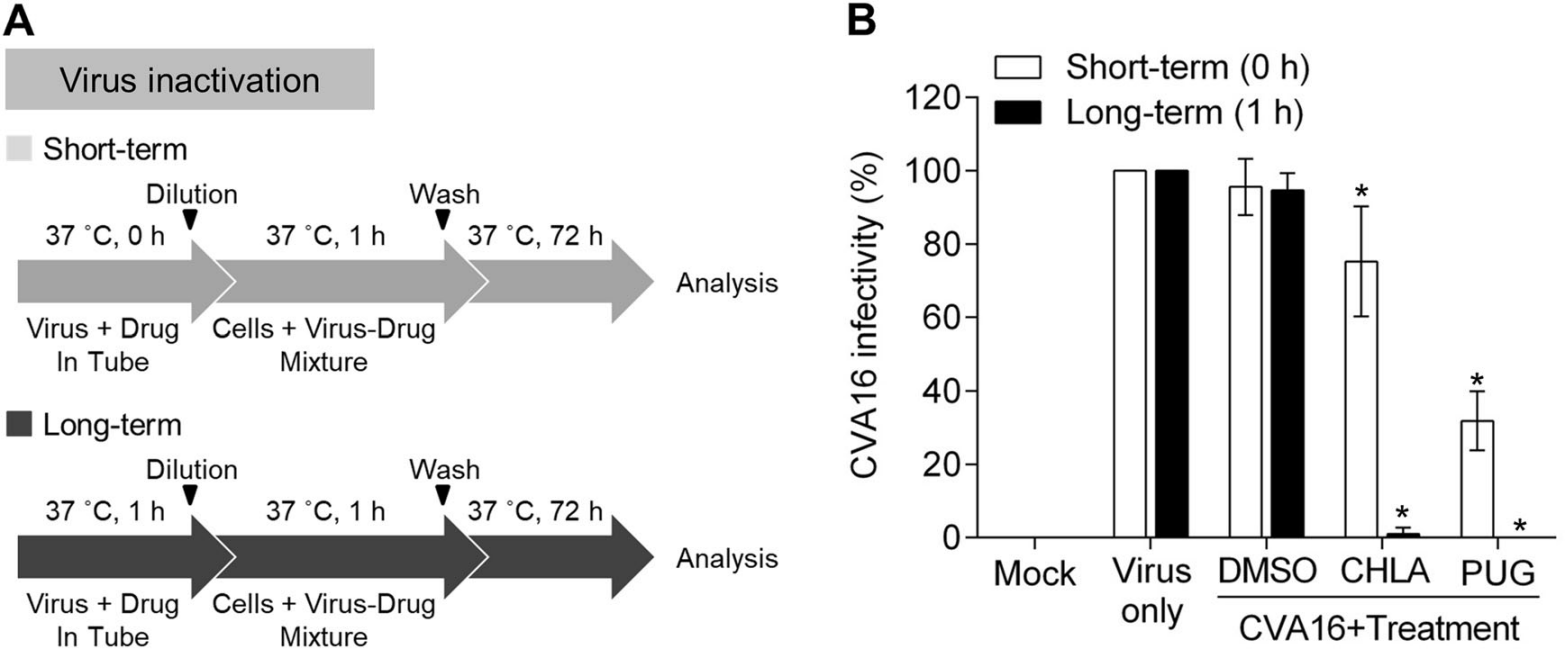
CHLA and PUG inactivate cell-free CVA16 virus particles. **a** Schematics of the experiment. **b** CVA16 (10^6^ PFU/ml) was treated with CHLA (20 µM) or PUG (25 µM) and incubated for 1 h at 37 °C, or were mixed immediately (0 h), and then diluted 50-fold to non-effective concentrations of the test compounds before inoculating the RD cells (final virus concentration = 50 PFU/well) and being analyzed for viral infectivity by a plaque assay. DMSO (0.25%) served as a negative control. The data shown are the means ± SD from three independent experiments. *p < 0.05 compared to the respective ‘virus only’ group

As CHLA and PUG inhibit viral binding (Fig. 3) with a minimum impact on pretreatment of the host cells (Fig. 2a), we speculated that the two tannins may interact with the viral particles as their primary target of inhibition. To investigate this possibility, we performed a viral inactivation assay^22^. Under cell-free conditions, the test compounds were preincubated with the CVA16 particles for 1 h (long-term) or directly mixed without incubation (short-term), after which they were diluted to a subtherapeutic concentration prior to inoculating RD cells (Fig. 4a). This dilution step prevents any meaningful interactions between the diluted test compounds and the cell monolayer. The resulting infection was subsequently analyzed by a plaque assay after 3 days of incubation. As shown in Fig. 4b, when the compounds were mixed with the virus and immediately diluted without incubation (short-term, ‘white bars’), CHLA and PUG inhibited ~20 and 70% of CVA16 infectivity, respectively, indicating that the two compounds can directly inactivate cell-free CVA16 particles upon contact. Prolonged exposure of the virus with the tannins further increased their antiviral effects, as demonstrated by the near complete loss of infectivity from the CVA16 inoculum (Fig. 4b; long-term, ‘black bars’). In contrast, treatment with DMSO resulted in viral infection in both scenarios. These observations indicated that the two tannins possess affinity for CVA16 particles and can inactivate them during the cell-free incubation, leading to irreversible neutralization of the viral infectivity. Taken together, our data suggest that CHLA and PUG inhibit CVA16 infection by targeting early viral entry, specifically through inactivating cell-free CVA16 viral particles and preventing viral attachment onto the host cell surface.

### CHLA and PUG target CVA16 capsid proteins and mask the pocket entrance

**Fig. 5:**
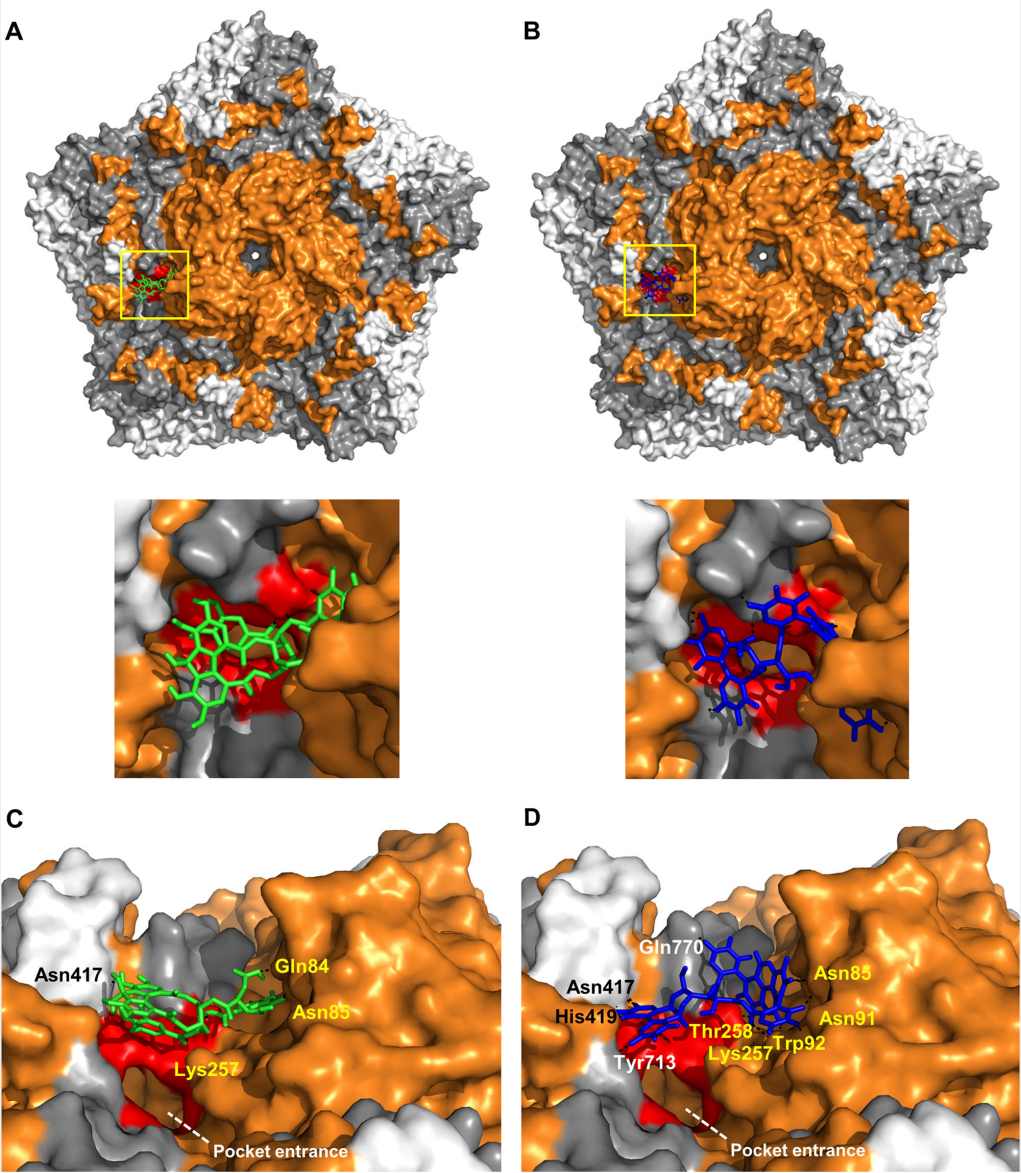
CHLA and PUG target the CVA16 capsid near the pocket entrance. **a**, **b** Molecular docking analysis of CHLA (**a**; green) and PUG (**b**; blue) on the pentamer of CVA16 (PDB: 5C4W); zoomed panels from the yellow demarcation are shown. VP1 = orange, VP2 = gray, and VP3 = white; polar contacts are shown as black dashes. Residues that make-up the pocket entrance are colored red (Ile^94^, Asp^95^, Gln^207^, Met^212^, Met^213^, Lys^257^, Thr^258^). **c**, **d** Alternate close-up side view into the canyon where CHLA and PUG bind near the pocket entrance. Unique residues from the polar contacts of the tannins to amino acids in VP1 (yellow font), VP2 (white font), and in VP3 (black font) are indicated. The white dashed line indicates the opening of the pocket region

The direct interaction between CHLA and PUG with the non-enveloped CVA16 particles led us to speculate that the tannins likely target the CVA16 capsid, including VP1, which harbors the pocket entrance for receptor binding^10^ and contains the epitopes for antibody neutralization^23^. To explore this hypothesis, we used a molecular docking analysis to predict the potential interaction(s) between the tannins and the CVA16 capsid pentamer. Both CHLA and PUG showed binding modes in the canyon region of the CVA16 pentamer as predicted by Autodock Vina, with CHLA having 6 polar contacts and a binding energy of 8.2 kcal/mol (Fig. 5a). PUG, which also bound to a similar site as CHLA, exhibited a stronger binding interaction, having 9 polar contacts and a binding energy of 12.3 kcal/mol (Fig. 5b). Specifically, both compounds bound directly above the pocket entrance (Fig. 5a, b, zoomed panels), which holds the pocket factor for mediating CVA16 entry. The unique residues from the polar contacts of the tannins surrounding the pocket entrance were determined for CHLA (VP1: Gln^84^, Asn^85^, and Lys^257^; VP2: Asn^417^; Fig. 5c) and PUG (VP1: Asn^85^, Asn^91^, Trp^92^, Lys^257^, Thr^258^; VP2: Asn^417^ and His^419^; and VP3: Gln^770^ and Tyr^713^; Fig. 5d), with most of these interactions occurring with VP1 for both compounds. As the pocket entrance is known to be crucial for SCARB2 interaction in displacing the pocket factor (e.g., sphingosine) to induce conformational change-dependent viral uncoating^10,24–26^, the ability of the tannins to bind to residues surrounding the pocket entrance suggests that they may potentially interfere with SCARB2 interaction and viral entry. This “masking” effect on the area for receptor binding and pocket factor displacement corroborate the inhibitory effect of the tannins against CVA16 attachment (Fig. 3) and in neutralizing viral particle infectivity (Fig. 4). These observations supports the greater overall antiviral activity of PUB compared to CHLA, due to its higher binding capacity. Altogether, the above results suggest that CHLA and PUG likely inhibit virus-receptor interactions and the associated viral entry mechanism via the identified CVA16 capsid residues.

## Discussion

Current management of CVA16 infections remains supportive, with good hygiene measures indicated as a means of prevention. While potential antiviral agents against the related EV71 virus have been explored, including pleconaril, metrifudil, N6-benzyladenosine, and NF449^27,28^, few have been tested or observed to exhibit inhibitory activity against CVA16^29,30^. In addition, despite ongoing vaccine development using CVA16 virus-like particles^31^ and inactivated viruses^32^, no approved prophylactic procedures exist to prevent large-scale epidemics. These issues underscore the imperative need to develop preventive and therapeutic strategies, including identifying effective antivirals against CVA16 for the management of the disease^5^. Our discovery that two tannins, CHLA and PUG, can inhibit CVA16 binding and efficiently disrupt CVA16 infection through targeting the viral capsid highlights the importance of entry inhibitors, which have been suggested to be a promising class of antivirals against enteroviruses^33,34^.

Our viral inactivation assay results (Fig. 4) point to the ability of the assayed tannins to directly target and inactivate CVA16 particles, and our molecular docking analysis predicted the binding sites of these tannins to be the VP1 pocket entrance in the canyon region (Fig. 5). The targeting of the pocket entrance by CHLA and PUG to inhibit CVA16 infection makes these two natural compounds highly unique and highlights their polyphenolic structures as important leads for blocking CVA16 infectivity. Interestingly, the recently identified capsid binders of the pyridyl imidazolidinone class, such as GPP3 and NLD, also target the hydrophobic pocket region of the enterovirus structural pentamer^34,35^. However, these imidazolidinone derivatives differ from the tannins described in this study, both structurally, and likely, functionally. GPP3 and NLD have a long chain-like structure that is similar to the hydrocarbon chain of the lipid pocket factor sphingosine^35^, whereas both CHLA and PUG are bulky polyphenolic structures consisting of gallic acid and hexahydroxydiphenoyl esters conjugated to a glucose pyranose core^36,37^. Due to their shapes and a higher binding energy, GPP3 and NLD exert antiviral activity by displacing the pocket factor and preventing structural conformational changes of VP1 from occurring during enterovirus entry^34^. In contrast, because the assayed tannins do not bury themselves in the hydrophobic pocket, we do not anticipate that they displace the pocket factor. Rather, the tannins are predicted to interact with several residues surrounding the pocket entrance and thereby “masking” it, which can potentially lead to a block in CVA16-receptor engagement, including SCARB2^10^. As such, the ability of CHLA and PUG to act as CVA16 capsid blockers provides structural information that could help in the development of antiviral bioactives for masking the pocket entrance and interfering with CVA16 entry. In addition, the identified residues (including Asn^85^, Lys^257^, and Asn^417^) that are targeted by both tannins in their polar contacts on the CVA16 capsid pentamer could also be further explored as potential neutralizing epitopes for developing CVA16 intervention strategies.

We previously observed that CHLA and PUG possess broad-spectrum antiviral activities in blocking the entry of viruses known to engage cell surface glycosaminoglycans (GAGs) as attachment factors, and we proposed that their antiviral mechanism was mediated through competing virus-GAG interactions^18,19^. Recently, heparan sulfate (HS) mimetics of cell surface GAGs have been shown to inhibit CVA16 infection, suggesting a potential role of GAGs in mediating CVA16 entry^21,38^. Interestingly, the binding locations of the tannins on the CVA16 capsid are also proximal to several clusters of positively charged amino acids that have been proposed to be the HS-interacting regions for the EV71 and CVA16 VP1 proteins^21,39^ (data not shown). Whether the targeting of CHLA and PUG for CVA16 VP1 competes with the function of GAGs as a binding factor in CVA16 entry remains to be elucidated. In addition, we cannot rule out that the tannin compounds are involved in additional modes of action, such as capsid destabilization and virion aggregation, which could also contribute to their antiviral activity against CVA16. A preliminary analysis using sucrose gradient ultracentrifugation of CVA16 particles showed a shift of the virus band to higher density virus fraction in the presence of the tannins (Supplementary Fig. S1), indicating potential virion aggregation, which can also affect virion binding to host cells. Further studies using mass spectrometry of banded virus fractions^40^, capsid stabilization analysis^35^, and surface plasmon resonance assay detection of ligand-drug binding^41^ could help characterize the biophysical interactions between the tannins and the CVA16 particle to elucidate the mechanism(s) of action of the compounds.

In summary, we identified the tannins CHLA and PUG as potent inhibitors of CVA16 entry. Future studies could also include structural modification assays to decrease their cytotoxicity and enhance their selectivity indices. Preliminary experiments indicate that both tannins also exhibit antiviral activity against EV71 infection (data not shown). We suggest that these natural compounds may have value for further development as candidate agents or as antiviral leads for the treatment and prevention of large-scale CVA16-induced HFMD epidemics.

## Materials and methods

### Cells and viruses

RD cells (ATCC#CCL-136; a gift from Dr. Shin-Ru Shih, Chang Gung University, Taiwan) were cultured in DMEM (GIBCO-Invitrogen; Carlsbad, CA, USA) supplemented with 10% fetal bovine serum (FBS) and a 1% penicillin-streptomycin and amphotericin B solution (Biological Industries; Beit Haemek, Israel). The clinical isolate of CVA16 was obtained from the Clinical Virology Laboratory at the Chang Gung Memorial Hospital (Taoyuan City, Taiwan). The virus was propagated in RD cells and viral titers were determined using a standard plaque assay as previously described^42^. For all infectivity assays, the basal medium consisted of DMEM containing 2% FBS and antibiotics.

### Test compounds

The assayed test compound classes, including tannins, triterpenoids, and synthetic derivatives of flavonoids and quinones, are listed in Table 1. CHLA, chebulinic acid, and PUG were purified from Fructus Chebulae as previously described^43,44^. 5-O-galloyl-shikimic acid was isolated from *Terminalia catappa L.*^45^ and was a gift from Dr. Ta-Chen Lin (Central Taiwan University of Science and Technology, Taiwan). The structure and purity of each compound was confirmed by high-performance liquid chromatography coupled with UV detection and electrospray ionization mass spectrometry (HPLC-UV/ESI-M) and HPLC with photodiode array detection (HPLC-PDA), respectively, as previously reported^46,47^. Saikosaponin a, b2, and c were purchased from Wako Inc. (Wako Pure Chemical Inc. Ltd.; Osaka, Japan). Synthetic derivatives of flavonoids (TCH-3105, TCH-3154, TCH-3188, TCH-3529, and TCH-3531) and quinones (Q4224, Q4236, Q4246, Q4248, and Q4250) were gifts from Dr. Chih-Hua Tseng (Kaohsiung Medical University, Taiwan). All compounds were dissolved in dimethyl sulfoxide (DMSO) and diluted with cell culture medium before use. The final concentration of DMSO in the drug solutions was equal to or below 0.25% for the experiments and a DMSO (0.25%) control was included in each analysis.

### Cytotoxicity assay

The cytotoxicity of the test compounds against RD cells (1 × 10^4^ cells/well of a 96-well plate) following a 3 day treatment were analyzed using an XTT (2,3-*bis*[2-methoxy-4-nitro-5-sulfophenyl]-5-phenylamino)-carbonyl]-2*H*-tetrazolium hydroxide)-based cell viability assay kit (Sigma; St. Louis, MO, USA) as previously reported to determine the 50% cytotoxic concentration (CC_50_)^18^.

### Drug screening and antiviral plaque assay

RD cells seeded in 12-well plates (2 × 10^5^ cells/well) were pretreated with the test compounds for 4 h. The cells were washed and challenged with CVA16 (50 plaque-forming units [PFU]/well) in the presence of the test compounds for 1 h. Following the infection, the wells were washed with PBS once, and the cells were subsequently overlaid with basal medium containing the test compounds and 0.8% methylcellulose (Sigma) before further incubation at 37 °C for 3 days. After the incubation, the cells were washed with PBS, fixed with 37% formaldehyde, and then stained with crystal violet (Sigma) to visualize viral plaques. The resulting CVA16 infection (%) was calculated as follows: (Mean # of plaques _virus+drug_/Mean # of plaques _virus+DMSO control_) × 100%. The concentration that reduced 50% of the plaque formation was defined as the ‘EC_50_’. The selectivity index (SI) of the test compounds was calculated as follows: SI = EC_50_/CC_50_.

### Time-of-drug-addition assay

The effect of drug addiction at various times during the viral infection process was assessed according to a previously described method with some modifications^18^. To evaluate the pretreatment effect of drugs, RD cells seeded in 12-well plates (2 × 10^5^ cells/well) were treated with CHLA (20 µM) or PUG (25 µM) for 1 or 4 h, after which the cells were washed with PBS before being challenged with CVA16 (50 PFU/well) in basal medium for 1 h. To assess the effect of adding the drugs and the virus concurrently (coaddition), RD cells were treated with CVA16 (50 PFU/well) and CHLA (20 µM) or PUG (25 µM) simultaneously for 1 h, after which the cells were washed and overlaid with media containing 0.8% methylcellulose. To study the drug treatment effect after viral entry, RD cells were challenged with CVA16 (50 PFU/well) for 1 h, and after the viral inoculum was removed, the infected cells were washed and overlaid with media containing CHLA (20 µM) or PUG (25 µM) plus 0.8% methylcellulose. For all experiments, viral plaques were stained with crystal violet and counted after a 72 h post infection incubation.

### Viral inactivation assay

The viral inactivation assay was performed as described previously with some modifications^18,22^. CVA16 (10^6^ PFU/ml) was mixed with CHLA (20 µM) or PUG (25 µM) and incubated at 37 °C for 1 h. The drug-virus mixture was subsequently diluted 50-fold with basal medium to a ‘subtherapeutic’ (i.e., ineffective) concentration of the compounds, which was subsequently added to RD-cell monolayers (2 × 10^5^ cells/well) seeded in 12-well plates (final virus concentration, 50 PFU/well). For a comparison, the drug-virus mixture was immediately diluted 50-fold (no incubation period) and added directly to the RD cells for infection. After incubation for 1 h, the diluted inocula were discarded and the cells were washed with PBS twice. Cells were subsequently overlaid with media containing 0.8% methylcellulose and incubated at 37 °C for 3 days before the plaque assay assessment as described above.

### Flow cytometry-based viral binding assay

Viral attachment to cell surfaces was assessed at 4 °C, which allows for viral binding but precludes entry for many viruses, including enteroviruses^39^. RD-cell monolayers (2 × 10^5^ cells/well in 12-well plates) were infected with CVA16 (multiplicity of infection [MOI] = 100) in the presence or absence of CHLA (20 µM), PUG (25 µM), soluble heparin (500 µg/ml; Sigma), or a DMSO control (0.25%) for 3 h at 4 °C. The viral inocula were subsequently removed, and the cells were collected into tubes by rinsing with ice-cold flow cytometry buffer (1X PBS plus 2% FBS). After cells were washed twice with ice-cold flow cytometry buffer and fixed with 4% paraformaldehyde for 20 min on ice, the cells were stained with an anti-VP1 antibody (1:2000; Merck-Millipore, Bedford, MA, USA) followed by incubation with a secondary Alexa 488-conjugated anti-mouse IgG (1:250, Invitrogen). Flow cytometry analysis was performed using a BD FACScan flow cytometer (Becton Dickinson; San Jose, CA, USA).

### Molecular docking analysis

The CVA16 mature virion crystal structure (PDB:5C4W)^48^ was determined using the Dock Prep module in the UCSF Chimera package^49^ developed by the Resource for Biocomputing, Visualization, and Informatics at the University of California, San Francisco (supported by NIGMS P41-GM103311). Solvents were deleted from the PDB file, incomplete side chains were replaced using the Dunbrach 2010 Rotamer Library, and hydrogens and charges were added to the structure according to previously described method^50^. The initial low energy 3D conformations of CHLA and PUG were created by taking the SMILES sequences from Pubchem (CID#442674 and #44584733, respectively) and then were generated using CORINA Classic (Molecular Networks GmbH, Germany and Altamira, LLC, USA). Molecular docking was performed using Autodock Vina (Scripps, LLC; La Jolla, CA, USA) to predict the binding mode and relative free energy of binding of the compounds to the protein target^51^. To confirm the results from the blind docking, which selects the whole protein as the docking site, docking was further confined to the outward surface of the viral protein in a 100 × 100 × 100 Å search box. Bound conformations were examined using the PyMol Molecular Graphics System (Version 1.7.4, Schrödinger, LLC; Portland, OR, USA).

### Statistical analysis

The data are presented as the means ± standard deviation (SD) from three independent experiments. Statistical significance was examined using the one-way analysis of variance (ANOVA) for comparing three or more groups. A *p-*value of <0.05 was considered significant.

## Electronic supplementary material

Supplementary information
