# Supplementary material for: Small molecules targeting coxsackievirus A16 capsid inactivate viral particles and prevent viral binding
Source: Emerg Microbes Infect. 2018 Sep 26;7:162. doi: 10.1038/s41426-018-0165-3 (PMC6156566; doi:10.1038/s41426-018-0165-3)
Supplement: Supplementary file 1 — Supplementary information [file 41426_2018_165_MOESM1_ESM.pdf]

Supplementary Information

Small Molecules Targeting Coxsackievirus A16 Capsid Inactivate Viral Particles and Prevent Virus Binding

Chien-Ju Lin[1], Ching-Hsuan Liu[2,3], Jonathan Y. Wang[4], Chun-Ching Lin[1,5], Yi-Fang Li[5], Christopher D. Richardson[3,6], and Liang-Tzung Lin[2,7,#]

[1] School of Pharmacy, College of Pharmacy, Kaohsiung Medical University, Kaohsiung 80708, Taiwan

[2] Graduate Institute of Medical Sciences, College of Medicine, Taipei Medical University, Taipei 11031, Taiwan

[3] Department of Microbiology & Immunology, Dalhousie University, Halifax, Nova Scotia B3H 4R2, Canada

[4] Department of Molecular Biosciences, The University of Texas at Austin, Austin, Texas 78712, United States of America

[5] Graduate Institute of Natural Products, College of Pharmacy, Kaohsiung Medical University, Kaohsiung 80708, Taiwan

[6] Department of Pediatrics and Canadian Center for Vaccinology, Izaak Walton Killam Health Centre, Halifax, Nova Scotia B3K 6R8, Canada

[7] Department of Microbiology and Immunology, School of Medicine, College of Medicine, Taipei Medical University, Taipei 11031, Taiwan

[#]CORRESPONDING AUTHOR

Liang-Tzung Lin, Department of Microbiology and Immunology, School of Medicine, College of Medicine, Taipei Medical University, No. 250, Wu-Hsing Street, Taipei 11031, Taiwan, Tel: +886-2-2736-1661 ext. 3911, Fax: +886-2-2736-1661 ext. 3921, E-mail: ltlin@tmu.edu.tw.

Supplementary Figure

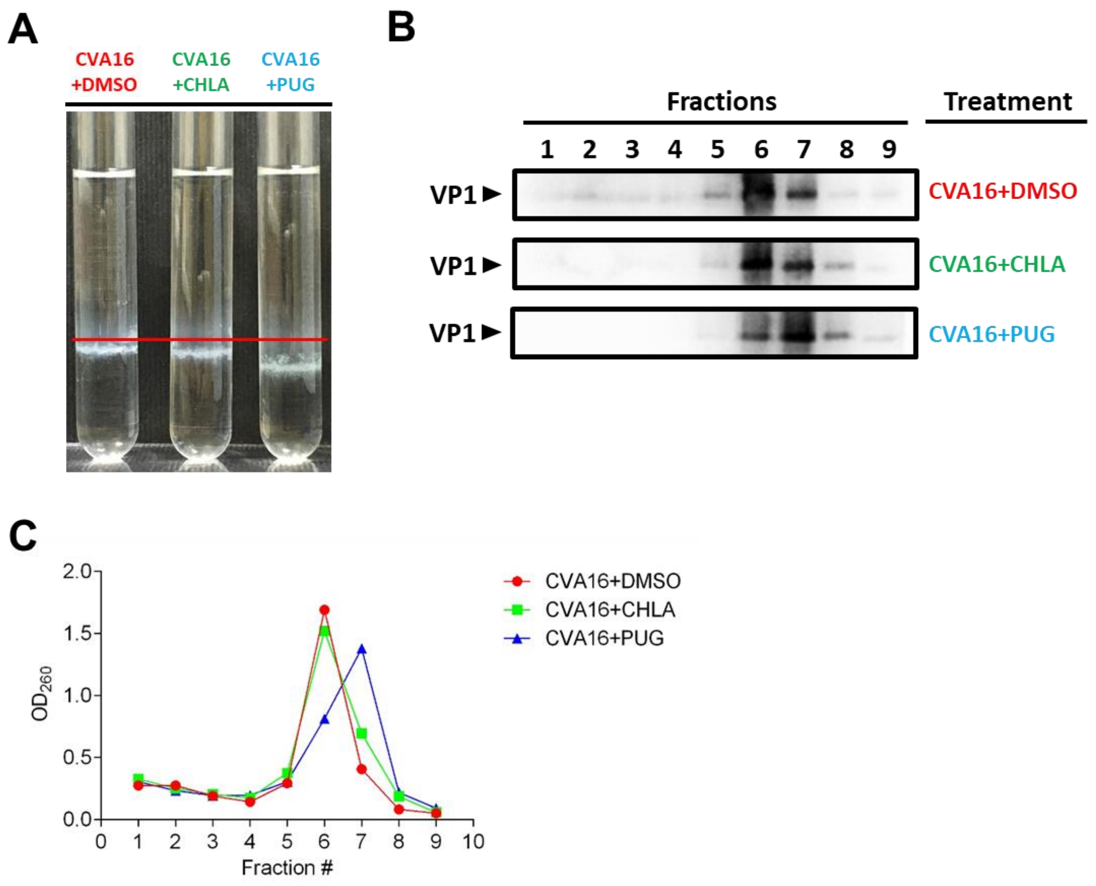

Fig. S1. Ultracentrifugation analysis of CVA16 particles treated with the tannins.
Polyethylene glycol-8000 (PEG8000)-concentrated CVA16 virions were treated with DMSO (0.25%), CHLA (20 µM) or PUG (25 µM) for 1 h at 37 °C. The drug-treated virus samples were layered onto a 20% to 60% sucrose gradient in 50 mM Tris-HCl (pH = 7), and then subjected to ultracentrifugation in a Beckman SW40 Ti rotor (Beckman Coulter; Brea, CA, USA) at 35,000 rpm for 3 h at 4 °C. Sedimentation of the virions at the end of the ultracentrifugation run is shown (**a**). Nine fractions (1 ml/fraction) from the ultracentrifugation were subsequently taken from top to bottom and subjected to Western blot analysis (**b**) and optical density assessment (**c**). For Western blot analysis, 18 µl of the fractions were subjected to SDS-PAGE and then transferred to PVDF membrane, which was blocked with 5% BSA for 1 h; the membrane was subsequently probed using anti-VP1 primary antibody (1:2000; Millipore) and anti-mouse HRP-conjugated secondary antibody (1:2000; Invitrogen) before detecting and imaging immunoreactive proteins using enhanced chemiluminescence reagent (Western Lightning ECL Pro; PerkinElmer, Waltham, MA, USA) and a Bio-Rad ChemiDoc Imager (Bio-Rad; Hercules, CA, USA). For measurement of optical density, individual fractions from the ultracentrifugation were diluted 5 times using ddH_2O and then analyzed using a spectrophotometer (Eppendorf; Hamburg, Germany) at 260 nm.
